# Repurposing COVID-19 Compounds (via MMV COVID Box): Almitrine and Bortezomib Induce Programmed Cell Death in *Trypanosoma cruzi*

**DOI:** 10.3390/pathogens14020127

**Published:** 2025-02-01

**Authors:** Carlos J. Bethencourt-Estrella, Atteneri López-Arencibia, Jacob Lorenzo-Morales, José E. Piñero

**Affiliations:** 1 Instituto Universitario de Enfermedades Tropicales y Salud Pública de Canarias, Universidad de La Laguna, Avda. Astrofísico Fco. Sánchez, S/N, 38203 La Laguna, Spain; cbthene@ull.edu.es (C.J.B.-E.); jmlorenz@ull.edu.es (J.L.-M.); jpinero@ull.edul.es (J.E.P.); 2 Departamento de Obstetricia y Ginecología, Pediatría, Medicina Preventiva y Salud Pública, Toxicología, Medicina Legal y Forense y Parasitología, Universidad de La Laguna, 38203 La Laguna, Spain; 3 Centro de Investigación Biomédica en Red de Enfermedades Infecciosas (CIBERINFEC), Instituto de Salud Carlos III, 28220 Madrid, Spain

**Keywords:** *Trypanosoma cruzi*, chemotherapy, apoptosis, COVID Box, MMV

## Abstract

Chagas disease, caused by the protozoan *Trypanosoma cruzi*, affects millions globally, with limited treatment options available. Current therapies, such as benznidazole and nifurtimox, present challenges, including their toxicity, side effects, and inefficacy in the chronic phase. This study explores the potential of drug repurposing as a strategy to identify new treatments for *T. cruzi*, focusing on compounds from the Medicines for Malaria Venture (MMV) COVID Box. An initial screening of 160 compounds identified eight with trypanocidal activity, with almitrine and bortezomib showing the highest efficacy. Both compounds demonstrated significant activity against the epimastigote and amastigote stages of the parasite and showed no cytotoxicity in murine macrophage cells. Key features of programmed cell death (PCD), such as chromatin condensation, mitochondrial membrane potential disruption, and reactive oxygen species accumulation, were observed in *T. cruzi* treated with these compounds. The potential to induce controlled cell death of these two compounds in *T. cruzi* suggests they are promising candidates for further research. This study reinforces drug repurposing as a viable approach to discovering novel treatments for neglected tropical diseases like Chagas disease.

## 1. Introduction

Protozoa from the *Trypanosoma cruzi* genus are the causative agents of Chagas disease, a significant parasitic illness predominantly affecting mainly Latin America. Recognized by the WHO as a critical global health concern, Chagas disease impacts approximately 6–7 million people worldwide, with considerable morbidity and mortality rates among tropical diseases [1,2]. The disease is intricately linked to factors such as poverty, inadequate housing, and limited access to healthcare, disproportionately affecting marginalized populations. Chagas disease manifests in two primary clinical phases, acute and chronic, each with distinct symptoms and severity, depending on the host’s immune response and the infection stage [3].

Current treatments for Chagas disease primarily involve the use of benznidazole and nifurtimox, which are most effective during the acute phase of the infection. However, these treatments come with significant drawbacks, including severe side effects, lengthy treatment regimens, and limited efficacy in the chronic phase of the disease. Additionally, the emergence of drug-resistant strains and the toxicity of existing medications further complicate the treatment landscape [4,5]. These challenges underscore the urgent need for novel therapeutic strategies to combat Chagas disease effectively.

In response to these hurdles, there is growing interest in exploring alternative approaches to treat Chagas disease. One promising strategy involves drug repurposing, which leverages existing drugs with established safety profiles and known pharmacokinetics to expedite the development of new treatment options for Chagas disease [6]. Drug repurposing offers several advantages, such as significantly reducing the time and cost associated with bringing new treatments to market and potentially uncovering new mechanisms of action against *Trypanosoma cruzi*.

The Medicines for Malaria Venture (MMV) has assembled the COVID Box, a collection of 160 compounds with known activity against SARS-CoV-2. Surprisingly, these compounds have demonstrated potential not only against the novel coronavirus but also against a variety of other infectious agents, including protozoan parasites like *L. braziliensis, L. amazonensis,* or *N. fowleri,* among others [7,8,9]. This study aims to screen and evaluate the efficacy of these compounds against *T. cruzi*, contributing to the search for better treatment options for Chagas disease.

When considering antiparasitic drugs, the type of cell death induced within the parasite is crucial. Necrotic death can lead to inflammation and tissue damage due to the release of various substances, whereas apoptotic death is a more controlled process that minimizes the immune response and tissue damage. Observing programmed cell death (PCD) or apoptosis-like processes in *T. cruzi* reveals several morphological changes, including cytoplasmic condensation, reduced cell volume, diminished mitochondrial membrane potential, chromatin condensation, and DNA fragmentation [10,11]. Apoptotic cells expose phosphatidylserine (PS) on their surface, facilitating recognition and phagocytosis by immune cells like macrophages, thereby reducing inflammation and tissue damage [12].

This article provides a comprehensive overview of the rationale behind drug repurposing, highlighting the advantages and challenges associated with this approach. It also outlines the methodologies used in screening and evaluating the efficacy of known drugs against *T. cruzi*, offering insights into their potential mechanisms of action and relevance in combating Chagas disease. Our findings aim to contribute to the growing body of knowledge in the quest for innovative and effective treatments for Chagas disease, offering renewed hope in the fight against this neglected tropical disease.

## 2. Materials and Methods

### 2.1. Chemicals

The compounds, stored at −20 °C, were obtained diluted in DMSO at 10 mM. Detailed data on the pathogen box compounds can be found at https://www.pathogenbox.org (accessed 30 May 2021). After initial screening, pure active compounds, bortezomib and almitrine mesylate, were purchased from Sigma-Aldrich (now Merck, Darmstadt, Germany).

### 2.2. Culture Cells

For the experiments, epimastigotes of *Trypanosoma cruzi* strain Y were cultured in the LIT (Liver Infusion Tryptose) medium, supplemented with 10% fetal bovine serum at 26 °C, and were grown to the log phase. Murine macrophages J774A.1 (ATCC #TIB-67) were maintained in Dulbecco’s Modified Eagle Medium (DMEM) at 37 °C with 5% CO_2_ and used for cytotoxic activity assays.

### 2.3. Trypanocidal Activity

An initial screening of the 160 compounds against *Trypanosoma cruzi* epimastigotes was performed according to the instructions of the MMV foundation at 10 μM. The activity of the compounds was then evaluated using alamarBlue^®^ (Invitrogen/Life Technologies, Madrid, Spain) with 10^6^ promastigotes per well. The concentration of DMSO never exceeded 0.1% (*v*/*v*) so as to avoid affecting parasite proliferation or morphology. Subsequently, the EnSpire multimode plate reader (PerkinElmer, Waltham, MA, USA) was used to measure the fluorescence after 72 h. Compounds that showed greater than 90% inhibition were tested for IC_50_. Serial dilutions were made in 96-well plates and incubated with parasites for 72 h after the addition of alamarBlue^®^. The IC_50_ values were calculated with GraphPad 9.0 (Boston, MA, USA) based on the fluorescence measurements [13].

### 2.4. Cytotoxic Activity

The cytotoxicity of the selected compounds was evaluated using a protocol similar to the one employed for assessing leishmanicidal activity. This assay utilized murine macrophages. Following a 24 h incubation with the compounds, alamarBlue^®^ was added, and the CC_50_ values were determined [14].

### 2.5. Variations in ATP Levels

Changes in cellular ATP levels due to treatments were measured using the CellTiter-Glo assay (Promega, Madison, WI, USA). Promastigotes were incubated with the compound at IC_90_ for 24 h, and luminescence was measured using an EnSpire spectrophotometer [15]. This assay was performed in three independent experiments.

### 2.6. Mitochondrial Membrane Potential Disruption

The JC-1 Mitochondrial Membrane Potential kit (Cayman Chemical, Ann Arbor, MI, USA) was used following the manufacturer’s instructions. Parasites were treated with the compound at IC_90_ for 24 h and then stained with JC-1 dye [16]. Three independent experiments were conducted for this assay.

### 2.7. Reactive Oxygen Species (ROS) Observation

Intracellular ROS accumulation was assessed using the CellRox DeepRed fluorescent probe (Invitrogen). Epimastigotes were incubated with the compounds at IC_90_ for 24 h, followed by CellRox staining and observation under a fluorescent microscope. This assay was repeated three times independently, with hydrogen peroxide (Merck, Germany) at 600 μM for 30 min, and was used as a positive control for ROS induction [17].

### 2.8. Plasmatic Membrane Permeability

To evaluate membrane permeability changes, the SYTOX^®^ Green assay was conducted on the parasites. Promastigotes were exposed to the compound at IC_90_ and incubated at 26 °C for 24 h. SYTOX^®^ Green was added at 1 μM (Molecular Probes^®^, Thermo Fisher Scientific, Waltham, MA, USA) for 15 min in the dark. Subsequently, the fluorescence was measured using an EnSpire^®^ Multimode Plate Reader (Perkin Elmer, Waltham, MA, USA) at GFP (excitation at 489 nm, emission at 508 nm). This experiment was repeated three times on separate days. An increased fluorescence indicated dye binding to the promastigote DNA. A positive control was included by permeabilizing the cells with 0.1% Triton X-100 (Sigma-Aldrich, St. Louis, MO, USA, now Merck, Darmstadt, Germany) [18].

### 2.9. Chromatin Condensation Evaluation

Chromatin condensation in the treated parasites was assessed using the Invitrogen Chromatin Condensation/Dead Cell Apoptosis Kit with Hoechst 33342 and Propidium Iodide (PI). Parasites were incubated for 20 min at 26 °C in the dark. Observations were made using an EVOS fluorescence microscope, with DAPI (excitation at 350 nm, emission at 461 nm) for the Hoechst and RFP (excitation at 535 nm, emission at 617 nm) for the PI. The images were organized into three categories: low fluorescence for viable cells, intense blue for chromatin condensation (apoptosis), and bright red for cell death [19].

### 2.10. ADME-Tox Predictions

The SwissADME web tool (Swiss Institute of Bioinformatics, Lausanne, Switzerland) was used to calculate the physicochemical descriptors and predict *in sílico* ADME and toxicity parameters. This tool assesses pharmacokinetic properties, drug-likeness, and medicinal chemistry friendliness to support drug discovery (Swiss Institute of Bioinformatics, 2023) [20].

### 2.11. Statistical Analyses

Data are presented as the mean ± standard deviation (SD) from at least three independent experiments. The inhibitory concentrations (IC_50_ and CC_50_) were calculated via non-linear regression analysis with 95% confidence intervals. Statistical differences between means were evaluated using one-way analysis of variance (ANOVA) for three or more samples, followed by Tukey’s test for pairwise comparisons, performed with GraphPad 9.0 software. A *p*-value of <0.05 was considered significant for all analyses.

## 3. Results

### 3.1. Trypanocidal and Cytotoxic Activity

After the initial screening of the MMV COVID Box, we identified compounds that demonstrated trypanocidal activity against *T. cruzi* epimastigotes, showing 51% or greater inhibition at only 10 μM (Table 1). Of the 160 compounds tested, 8 met this criterion, with the majority showing inhibition ranging from 61–70% (4 compounds), and where none of them exceeded 80% inhibition. These active compounds are listed in Table 1, indicating the activity groups to which each one belongs.

After carrying out a literature search to find out if any of these compounds had been previously studied in *Trypanosoma cruzi* or other protozoa and then checking the availability to purchase the pure compounds, it was decided to buy the following: almitrine, bortezomib, and ABT239. After receiving them, weighing them, and making dilutions of them, trypanocidal activity assays were performed to obtain the inhibitory concentration of 50 of the compounds, both against the epimastigote and the amastigote phases of the parasite. In addition, their cytotoxicity against a mouse macrophage cell line was also determined and can be seen in Table 2.

As can be seen from the table, the compound data are consistent between the epimastigotes and amastigotes, with almitrine being the most active, followed by bortezomib. Furthermore, these two compounds showed no cytotoxicity against mouse cells. On the other hand, ABT239 did not show high activity on the epimastigotes and, therefore, was not evaluated against the amastigotes, as it was also slightly toxic against mouse macrophages.

After obtaining the activity and cytotoxicity values, we were able to obtain the ratio, which was indicative of the selectivity of the compounds, which are shown in Table 3. The higher the ratio, the greater the selectivity of the compound against the parasite.

The selectivity results on the amastigote stage indicate that almitrine has a selectivity greater than 120, while bortezomib has a selectivity higher than 90. None of the compounds outperformed the reference drug benznidazole, which has an index of around 150.

### 3.2. Plasmatic Membrane Permeability and Chromatin Condensation Evaluation

**Figure 1:**
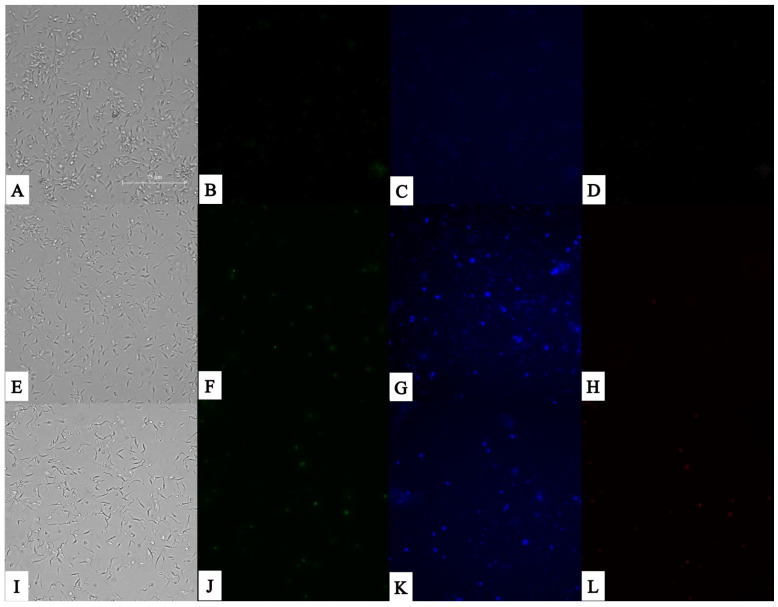
SYTOX^®^ Green nucleic acid stain fluorescent dye (ThermoFisher Scientific, Waltham, MA, USA) for the detection of plasmatic membrane permeability, and Vybrant^®^ Apoptosis Assay Kit n°5 (ThermoFisher Scientific, Waltham, MA, USA) for the detection of chromatin condensation. Results after 24 h of incubation with the IC_90_ of the compounds with epimastigote stage of *T. cruzi*. Images were captured using an EVOS FL Cell Imaging System (40×). (**A**) Parasites without treatment in visible channel; (**B**) parasites without treatment in GFP channel; (**C**) parasites without treatment in DAPI channel; (**D**) parasites without treatment in RFP channel; (**E**) parasites treated with almitrine in visible channel; (**F**) parasites treated with almitrine in GFP channel; (**G**) parasites treated with almitrine in DAPI channel; parasites treated with almitrine in RFP channel; (**H**) parasites without treatment in DAPI channel; (**I**) parasites treated with bortezomib in visible channel; (**J**) parasites treated with bortezomib in GFP channel; (**K**) parasites treated with bortezomib in DAPI channel; and (**L**) parasites treated with bortezomib in RFP channel. Scale bar: 75 μm.

Once the parasites were incubated with the IC_90_ of both study compounds, almitrine and bortezomib, we saw that neither of them induced significant permeabilization in the parasites after 24 h of incubation. This is well observed in the second column of Figure 1, where the parasites observed in the GFP channel appear, where, in green, the cells that have increased their permeability appear, which we visualized thanks to the binding of SytoxGreen to the cellular DNA.

On the other hand, in Figure 1, we can also observe how bortezomib is the compound that induces a greater increase in cells with condensed chromatin after 24 h of incubation. This is well observed in the third column, where the parasites observed in the GFP channel appear, where the cells that have condensed their chromatin appear in blue, and which we visualized thanks to the binding of Hoechst to the cellular DNA. In addition, we can even observe how, in the fourth column, bortezomib is the only one that induces a faster death in the parasites, evidenced by the penetration of propidium iodide in some parasites.

### 3.3. Variations in ATP Levels

**Figure 2:**
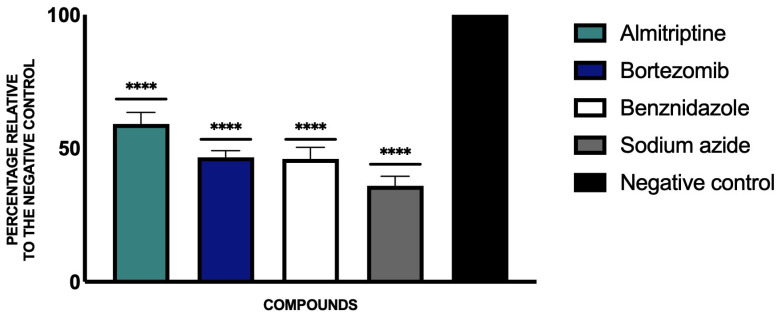
Percentage relative to the negative control of the results of ATP levels. Benznidazole was used as the reference treatment, and sodium azide was used as a positive control. A Tukey test with GraphPad.PRISM^®^ 9.0.0 software was used to test the statistical differences between means (**** *p* < 0.0001).

By using JC-1, we looked at the changes in the mitochondrial membrane potential of the parasite culture after incubation with the study compounds. We see, in Figure 2, how the two drugs, almitrine and bortezomib, induced a slight yet significant decrease in mitochondrial membrane potential compared to the untreated parasite control. To represent these results, the untreated control was taken as 100% ATP, so we see how almitrine and bortezomib reduce the ATP levels by about 50% compared to the control.

### 3.4. Mitochondrial Membrane Potential Disruption

**Figure 3:**
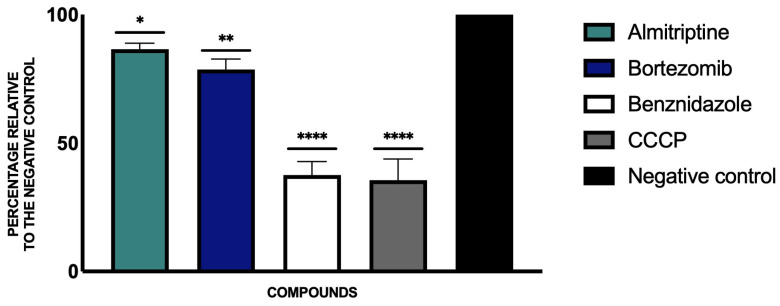
Results of mitochondrial membrane potential alterations expressed as percentages relative to negative control. Benznidazole was added as the reference treatment, and carbonyl cyanide m-chlorophenyl hydrazone (CCCP) was used as a positive control. A Tukey test with GraphPad.PRISM^®^ 9.0.0 software was used to test the statistical differences between means. (* *p* < 0.05; ** *p* < 0.01; and **** *p* < 0.0001).

After analyzing the changes in the mitochondrial membrane potential of the parasite culture after incubation with the study compounds, we saw how the two drugs, almitrine and bortezomib, induced a slight, although significant, decrease in the mitochondrial membrane potential compared to the untreated parasite control, as seen in Figure 3. To represent these results, the ratio between red (JC-1 dimer) and green (JC-1 monomer) fluorescence was performed, and the untreated control was taken as the highest value, characteristic of healthy cells.

### 3.5. Reactive Oxygen Species (ROS) Observation

When the parasites were incubated for 24 h with the study compounds, we observed that both compounds, as well as the positive control with H_2_O_2_, induced an increase in the accumulation of reactive oxygen species in the cell cytoplasm. This increase in ROS may be due to poor management by the cells in the detoxification of these species, and this could be, in the first instance, the phenomenon that triggers the cascade of apoptotic events.

**Figure 4:**
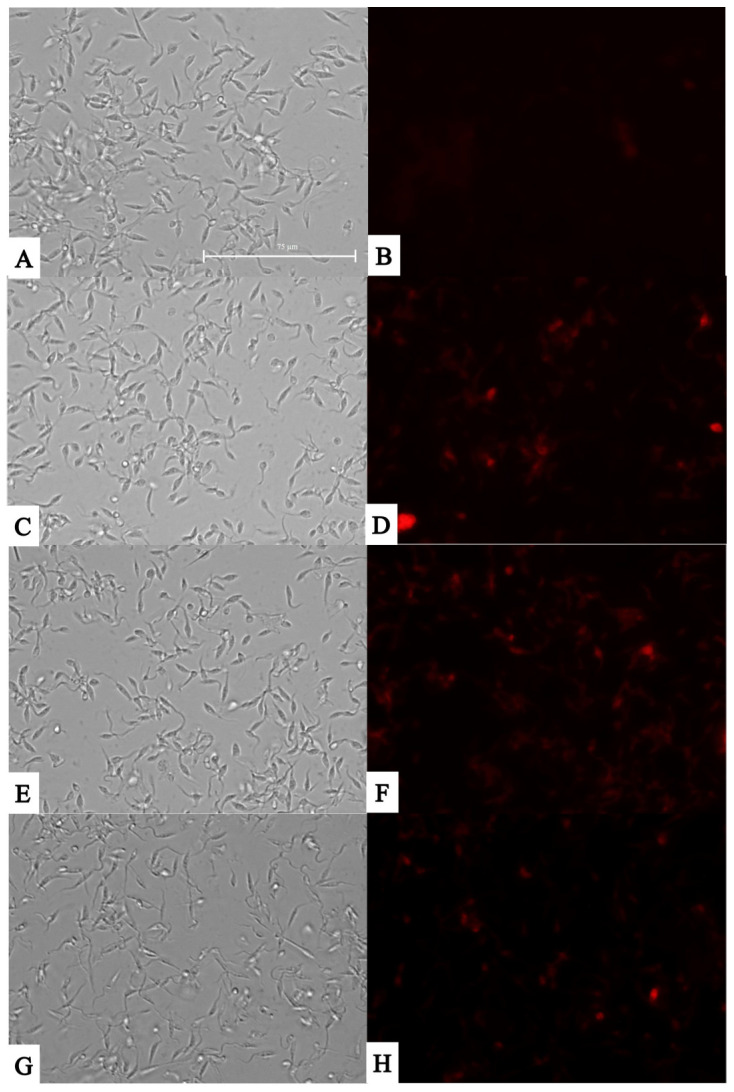
Presence of reactive oxygen species against epimastigote stage of *T. cruzi* using CellROX^®^ Deep Red staining (ThermoFisher Scientific, Waltham, MA, USA). Images were captured using an EVOS FL Cell Imaging System (40×). (**A**) Parasites without treatment in visible channel; (**B**) parasites without treatment in Cy5 channel; (**C**) parasites treated with H_2_O_2_ 600 mM in the visible channel; (**D**) parasites treated with H_2_O_2_ 600 mM in Cy5 channel; (**E**) parasites treated with almitrine in visible channel; (**F**) parasites treated with almitrine in Cy5 channel; (**G**) parasites treated with bortezomib in the visible channel; and (**H**) parasites treated with bortezomib in Cy5 channel. Scale bar: 75 µm.

It should be noted that, as can be seen in the transmitted light images in Figure 4, the parasites treated for 24 h with the study compounds continue to maintain their integrity, both in the shape of the parasite and its cell membrane, as well as its flagellum; therefore, a necrotic type of death could be totally excluded.

### 3.6. ADME/Tox Predictions

**Figure 5:**
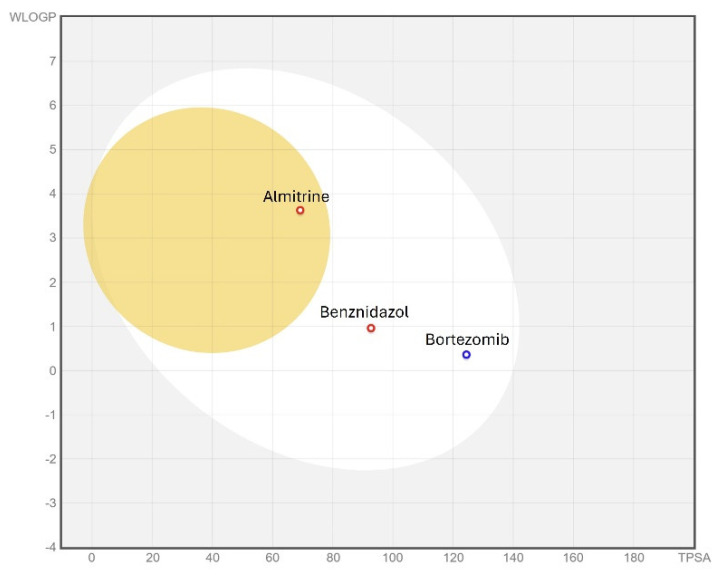
BOILED-Egg graph illustrating the *in sílico* prediction of human intestinal absorption (HIA) and blood–brain barrier (BBB) penetration. The white area represents a high likelihood of passive absorption in the gastrointestinal tract, while the yellow area indicates a high probability of brain penetration. Additionally, points are colored blue if the compound is predicted to be actively effluxed by P-glycoprotein (P-gp+) and red if predicted to be a non-substrate of P-gp (P-gp−).

When evaluating the ADME-Tox properties of a drug, a good starting point is to assess the drug’s gastrointestinal absorption and its ability to cross the blood–brain barrier (BBB), which are key pharmacokinetic properties during drug discovery. The Brain Or IntestinaL EstimateD (BOILED-Egg) permeation method is a reliable predictive model that estimates the lipophilicity and polarity of small molecules. For this reason, the BOILED-Egg prediction was applied to the selected compounds and bortezomib, as shown in Figure 5.

The egg graph indicates that the two compounds, along with benznidazole, are predicted to be well-absorbed at the intestinal level, and almitrine is, in addition, likely to cross the BBB. Moreover, bortezomib is predicted to be a non-substrate of P-glycoprotein (P-gp), unlike almitrine and benznidazole. Being a substrate of P-gp suggests that these compounds could be expelled from cells by this transmembrane pump. A drug that does not bind to P-gp has several potential advantages: it may exhibit higher bioavailability, and it might reduce drug–drug interactions since non-binding drugs are less likely to interact with others that do [21].

The bioavailability radar, which was also performed for each compound, provides a quick assessment of six physicochemical properties that predict drug bioavailability, crucial for understanding its pharmacokinetics. Generally, the selected compounds show parameters within optimal ranges, but almitrine slightly exceeds the optimal size and lipophilicity, and bortezomib has excess molecular flexibility.

SwissADME also provides access to five different rule-based filters that are designed to evaluate whether molecules possess oral drug-like properties based on the specific property ranges. These filters originate from research conducted by major pharmaceutical companies aiming to improve the quality of their proprietary chemical libraries. The first and most well-known rule-of-five method, the Lipinski (Pfizer) filter, was introduced with criteria that included lipophilicity, hydrogen bond donors and acceptors, molecular weight, and flexibility. Other rule sets, such as Ghose or Veber, use the same parameters but with other cut-off values [20].

**Figure 6:**
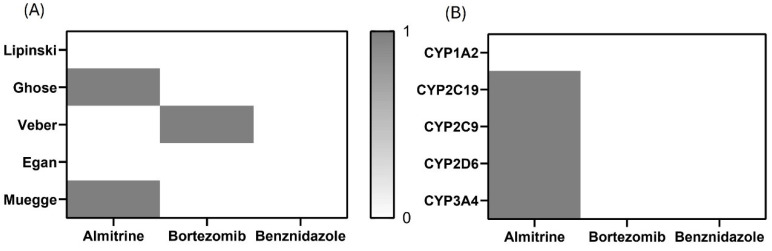
Heatmaps of (**A**) predicted drug-likeness, established as qualitatively the chance for a molecule to become an oral drug with respect to bioavailability, and (**B**) predicted cytochrome P450 (CYP) inhibition. White: no inhibition; gray: inhibition.

As shown in Figure 6A, benznidazole is the only compound that does not violate any of the five rule-based filters established by pharmaceutical companies for the qualitative prediction of drug-likeness in orally administered drugs. Bortezomib follows in second place, with only one violation of Veber’s rule. Almitrine comes next, showing two violations: one in the Ghose filter and another in Muegge’s rule.

A key concern in drug discovery, especially regarding toxicity, is avoiding the inhibition of cytochrome P450 (CYP) enzymes, which play a critical role in drug metabolism. Inhibiting CYP can reduce drug elimination, leading to drug–drug interactions (DDIs) and causing adverse effects; so, identifying potential CYP inhibition is therefore essential in drug development and clinical treatments [22]. The five major human CYP isoforms involved in drug metabolism are 1A2, 2C9, 2C19, 2D6, and 3A4, which together account for about 95% of the CYP-mediated drug metabolism, representing roughly 75% of the overall drug metabolism. In Figure 6B, bortezomib and benznidazole stand out as the compounds that do not inhibit any of the studied CYP isoforms in silico, while almitrine inhibits four of the five CYP enzymes studied.

## 4. Discussion

The present study screened the MMV COVID Box for compounds with trypanocidal activity against *Trypanosoma cruzi* epimastigotes. Out of the 160 compounds tested, 8 met the criterion of at least 51% inhibition at 10 μM. These active compounds include cycloheximide, almitrine, bortezomib, (-)-anisomycin, delanzomib, nitazoxanide, manidipine, and SAX-187, belonging to anti-infective agents, antitumor compounds, and agents affecting the nervous, cardiovascular, or respiratory systems.

After conducting a literature search to identify the compounds previously studied in *Trypanosoma cruzi* or other protozoa, we searched on the availability of pure compounds to purchase, such as almitrine, bortezomib, and ABT239. It is worth mentioning that since the literature already had studies on this drug regarding its activity against the *Trypanosoma cruzi* parasite [23], in our study, terconazole was discarded.

The IC_50_ of the compounds was determined against both the epimastigote and amastigote phases of the parasite. Additionally, cytotoxicity was assessed against a mouse macrophage cell line. Almitrine exhibited the highest activity, followed by bortezomib, both showing no cytotoxicity against murine cells. ABT239 did not demonstrate high activity against the epimastigotes and was slightly toxic to macrophages, so it was not evaluated against the amastigotes. The selectivity index for almitrine was greater than 120, and for bortezomib, it exceeded 90. However, none of the compounds outperformed the reference drug benznidazole, which has an index of around 150. In previous studies by our group, where these compounds were studied and also found to be active against other kinetoplastids, such as *Leishmania amazonensis* and *L. donovani*, we obtained different selectivity indexes, i.e., 2500 for bortezomib and 46 for almitrine for the *L. amazonensis* amastigotes; however, the activity in the amastigotes of almitrine in *L. amazonensis* (2.2 μM) was similar to that obtained in *T. cruzi* (close to 1.6 μM). However, bortezomib showed 100 times higher activity against the *L. amazonensis* amastigotes than against *T. cruzi* in the present work (0.04 μM vs. 2.79 μM).

An efficient treatment for Chagas disease must trigger controlled parasite elimination by initiating programmed cell death (PCD) in the parasite. In *Trypanosoma*, specific indicators of PCD include chromatin condensation, changes in plasma membrane permeability, elevated ROS production, and reduced mitochondrial membrane potential [24,25]. Conversely, unregulated cell death without typical apoptotic or autophagic features can provoke inflammatory responses, exacerbating tissue deterioration in infected patients. This damage primarily affects vital parasite-infected tissues (such as cardiac or gastrointestinal) due to the cytokine storm and cellular debris. Our study revealed that bortezomib and almitrine significantly decreased mitochondrial membrane potential in the treated parasites. In parallel, the two selected compounds caused an increase in chromatin condensation, often associated with early apoptosis, as well as an accumulation of reactive oxygen species within the epimastigotes. Moreover, the pharmacokinetic evaluation of the compounds reveals that benznidazole is the most favorable in terms of bioavailability and drug-likeness, with no rule violations, while almitrine shows potential issues with CYP enzyme inhibition and rule violations. Bortezomib, despite minor flexibility concerns, does not inhibit CYP enzymes, making it a promising candidate with minimal drug-drug interaction risks.

Additionally, given the broad-spectrum activity of the compounds evaluated, they are likely to exhibit significant efficacy against various pathogenic microorganisms. For example, bortezomib, a groundbreaking proteasome inhibitor, was approved by the FDA for the treatment of refractory or relapsed multiple myeloma [26]. It selectively and reversibly disrupts the ubiquitin-proteasome pathway, and its inhibition in the *Leishmania* genus is known to hinder parasite proliferation and differentiation, thereby affecting its virulence factor [27]. On the other hand, almitrine, a diphenylmethylpiperazine derivative, is classified as a respiratory stimulant and is used to treat hypoxemia caused by obstructive bronchitis. It improves respiration by acting as an agonist of peripheral chemoreceptors located on the carotid bodies, thereby stimulating these receptors and enhancing the respiratory process [28].

Several researchers have achieved encouraging results in previous studies using the MMV COVID Box to explore its biological activity against parasites. For instance, Dos Santos and colleagues tested the Box against *Toxoplasma gondii* and identified 29 compounds that were active against the parasite’s tachyzoites. Following *in sílico* predictions of the physicochemical properties and drug-likeness, they determined that the most promising compound for further research was almitrine [29]. This compound was also selected in the current study, underscoring its antiprotozoal potential.

Another study on the COVID Box in *Acanthamoeba* spp. performed the initial screening, and after obtaining the cytotoxicity results and evaluating the availability of drugs on the market, they selected two compounds: almitrine and terconazole. They identified the latter as the most active against different strains of *Acanthamoeba* [30], inducing programmed cell death (PCD) in the amoeba via the inhibition of sterol biosynthesis inhibition. Chao-Pellicer and Col., investigating the COVID Box, conducted a similar screening of activity against *Naegleria fowleri*, the brain-eating amoeba, evaluating the cytotoxicity as well as availability in the market. From this analysis, four compounds were selected: terconazole, clemastine, ABT-239, and PD-144418, which were identified as the most effective against *N. fowleri* and induced programmed cell death (PCD) in the amoeba [9].

Moreover, in previous work by our group studying the COVID box, we identified four compounds with high leishmanicidal activity, being almost the same as those found in the present study (bortezomib, almitrine, terconazole, and ABT239), with bortezomib standing out as being 100 times more selective than miltefosine. Furthermore, these compounds were shown to induce programmed cell death in *Leishmania amazonensis* [8]. In addition, in another study, the *in vitro* activity of bortezomib was tested against blood forms of *Trypanosoma brucei*, obtaining a high activity (IC_50_ of 3.3 nM) and highlighting its trypanocidal capacity [31]. Therefore, the trypanocidal capacity of bortezomib and almitrine has been demonstrated, and the activity of almitrine in strains causing sleeping sickness remains to be tested.

If we compare our results of trypanocidal activity with other compounds highlighted in the literature, we see that Posaconazole, Amiodarone, and Ravuconazole stand out as having significant efficacy against *T. cruzi*. For instance, Posaconazole demonstrates potent activity with an IC_50_ of 0.25 nM in amastigotes, outperforming many other drugs in its class. Similarly, Amiodarone has shown trypanocidal properties, particularly in disrupting calcium homeostasis within the parasite, with reported IC_50_ values ranging from 2.7 µM [32]. Ravuconazole, a broad-spectrum antifungal, has also displayed promising results, with an IC_50_ against the intracellular amastigote form of 0.1 nM, comparable to those of Posaconazole [33]. When compared to almitrine and bortezomib, as discussed in the manuscript, it is notable that almitrine’s IC_50_ against *T. cruzi* amastigotes is slightly higher (1.62 µM), while bortezomib’s activity (IC_50_ = 2.79 µM) remains within an effective range. These comparisons emphasize the need to balance efficacy with selectivity indices and cytotoxicity profiles to optimize therapeutic potential.

Finally, the fact that these compounds have different biological activities, such as anti-COVID, anti-trypanosome, anti-leishmanial, or anti-toxoplasma, might seem that they are very unspecific compounds, and therefore also toxic to human cells; however, they are drugs already marketed and used to treat different pathologies such as multiple myeloma or hypoxemia. It should be noted that, on the one hand, almitrine was withdrawn from the European market for causing neuropathies and weight loss [34], and, on the other hand, special care must be taken in the use of bortezomib, as it is an antineoplastic agent where the dose to be administered must be carefully evaluated without being harmful to the patient. In contrast, we consider that almitrine and bortezomib stand out for their capacity as broad-spectrum anti-infective agents since almitrine shows activity against amoebae of the genera *Acanthamoeba*, *Plasmodium falciparum*, *Leishmania amazonensis*, and now, *Trypanosoma cruzi*. Meanwhile, bortezomib has shown activity against *P. falciparum*, *Babesia divergens*, *Leishmania amazonensis, L. donovani*, *Trypanosoma brucei,* and now, *T. cruzi*, demonstrating its antiprotozoal capacity [8,29,30,31,35,36,37].

## 5. Conclusions

In conclusion, this study highlights the promising potential of drug repurposing as an effective approach for developing new treatments for neglected tropical diseases, specifically Chagas disease. The compounds almitrine and bortezomib, identified through screening the MMV COVID Box, demonstrated significant activity against both the epimastigote and amastigote stages of *Trypanosoma cruzi*. These compounds also induced key features of programmed cell death, such as chromatin condensation, mitochondrial membrane potential disruption, and reactive oxygen species accumulation. While neither compound outperformed the reference drug benznidazole, their trypanocidal activity, in addition to an antiprotozoal broad-spectrum capacity and low cytotoxicity, suggest they are viable candidates for further investigation, highlighting bortezomib as the most favorable in terms of bioavailability, drug-likeness, and toxicity. Although *in vivo* trials in appropriate animal models should be conducted to corroborate these results, this study underscores the importance of exploring novel therapeutic pathways to address the limitations of current Chagas disease treatments, offering hope for improved outcomes in managing this global health challenge.

## Figures and Tables

**Table 1:** Active compounds against *T. cruzi* from the COVID Box divided by their biological activity group.

Compound	Structure	Biological Activity Group	% of Inhibition
Cycloheximide	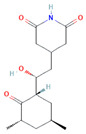	Fungicidal agent (agriculture)	61–70%
Almitrine	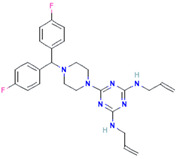	Respiratory system agent	61–70%
Bortezomib	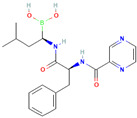	Antitumor agent (multiple myeloma)	61–70%
(-)-Anisomycin	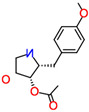	Anti-infective agent (antibiotic)	51–60%
Delanzomib	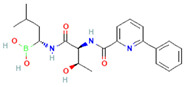	Antitumor agent (multiple myeloma)	51–60%
Nitazoxanide	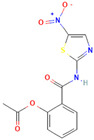	Anti-infective agent (antiparasitic)	61–70%
Manidipine	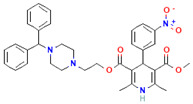	Cardiovascular agent (antihypertensive)	71–80%
SAX-187	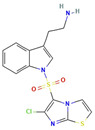	Nervous system agent (antipsychotic)	71–80%
ABT-239	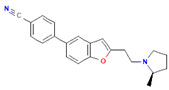	Nervous system agent	51–60%

**Table 2:** Results of *in vitro* activity against intracellular and extracellular forms of *T. cruzi* and cytotoxicity against murine macrophages. The results of IC_50_ and CC_50_ were expressed in mean ± standard deviation. Benznidazole was used as a reference treatment. All the results were expressed in μM for IC_50_: inhibitory concentration 50; CC_50_: cytotoxic concentration 50.

Compound	IC_50_ Epimastigote Stage of *T. cruzi*	IC_50_ Amastigote Stage of *T. cruzi*	CC_50_ Murine Macrophages
Almitrine	10.38 ± 0.42	1.62 ± 0.35	>200
Bortezomib	30.35 ± 1.30	2.79 ± 0.28	>250
ABT239	80.11 ± 0.91	-	68.48 ± 2.57
Benznidazole	6.92 ± 0.77	2.67 ± 0.39	399.91 ± 1.4

**Table 3:** Results of *in vitro* selectivity against intracellular and extracellular forms of *T. cruzi*. Benznidazole was used as a reference treatment. SI: selectivity index.

Compound	SI Epimastigote Stage of *T. cruzi*	SI Amastigote Stage of *T. cruzi*
Almitrine	>19.3	>123.5
Bortezomib	>8.2	>89.6
ABT239	0.9	-
Benznidazole	57.8	149.8

## Data Availability

The raw data supporting the conclusions of this article will be made available by the authors upon request.
